# Recycling Antibiotics into GUMBOS: A New Combination Strategy to Combat Multi-Drug-Resistant Bacteria

**DOI:** 10.3390/molecules20046466

**Published:** 2015-04-10

**Authors:** Marsha R. Cole, Jeffery A. Hobden, Isiah M. Warner

**Affiliations:** 1 Commodity Utilization, Southern Regional Research Center, Agricultural Research Services, United States Department of Agriculture, New Orleans, LA 70124, USA; 2 Department of Microbiology, Immunology, and Parasitology, Louisiana State University Medical Center, New Orleans, LA 70112, USA; 3 Department of Chemistry, Louisiana State University, Baton Rouge, LA 70803, USA

**Keywords:** chlorhexidine, β-lactam antibiotic, multi-drug resistant, GUMBOS, combination drug therapy, ion pair, antibacterial, synergy

## Abstract

The emergence of multi-drug-resistant bacteria, coupled with the lack of new antibiotics in development, is fast evolving into a global crisis. New strategies utilizing existing antibacterial agents are urgently needed. We propose one such strategy in which four outmoded β-lactam antibiotics (ampicillin, carbenicillin, cephalothin and oxacillin) and a well-known antiseptic (chlorhexidine di-acetate) were fashioned into a group of uniform materials based on organic salts (GUMBOS) as an alternative to conventional combination drug dosing strategies. The antibacterial activity of precursor ions (e.g., chlorhexidine diacetate and β-lactam antibiotics), GUMBOS and their unreacted mixtures were studied with 25 clinical isolates with varying antibiotic resistance using a micro-broth dilution method. Acute cytotoxicity and therapeutic indices were determined using fibroblasts, endothelial and cervical cell lines. Intestinal permeability was predicted using a parallel artificial membrane permeability assay. GUMBOS formed from ineffective β-lactam antibiotics and cytotoxic chlorhexidine diacetate exhibited unique pharmacological properties and profound antibacterial activity at lower concentrations than the unreacted mixture of precursor ions at equivalent stoichiometry. Reduced cytotoxicity to invasive cell types commonly found in superficial and chronic wounds was also observed using GUMBOS. GUMBOS show promise as an alternative combination drug strategy for treating wound infections caused by drug-resistant bacteria.

## 1. Introduction

Ineffective antibiotics are a pervasive reality that has contributed to higher incidences of infectious diseases caused by multi-drug-resistant bacteria, namely “ESKAPE” pathogens (*i.e*., *Enterococcus faecium*, *Staphylococcus aureus*, *Klebsiella pneumoniae*, *Acinetobacter baumannii*, *Pseudomonas aeruginosa* and *Enterobacter* species) [1]. The nonexistent antibiotic pipeline and perceived lack of political interest towards drug-resistant bacteria have worsened the fight against drug-resistant infections and has initiated a post-antibiotic era [2]. Thus, last resort treatment, particularly for drug-resistant superficial and chronic wound management, relies heavily on prompt and appropriate antimicrobial therapy for the delivery of improved clinical prognoses [3].

Drug-resistant bacteria represent an area of increasing concern in wound infections. Wound colonization with multi-drug-resistant bacteria requires aggressive treatment with the limited arsenal of effective, therapeutic antibiotics. Recent interest in counteracting drug-resistant wound infections has led to administering antibiotics simultaneously for treatment [3]. Combination drug therapy has contributed to some clinical and commercial successes against many vectors of human disease [4,5,6]. In particular, antibiotic combinations have been used to: (1) improve the efficacy of treatment; (2) reduce dosing concentrations; (3) extend treatment response; (4) expand pharmaceutical use of an approved drug; and (5) minimize the rate at which microbes acquire resistance [5]. In the last decade, however, research interest in antibiotic combinations has grown from the conventional use of two antibiotics to substituting one antibiotic with an unconventional over-the-counter consumer drug or prescription-only non-antibiotic drug. Examples of these efforts have expanded to include barbiturates, antipsychotics, antivirals and analgesics to improve the efficacy of antibiotic treatment [7,8,9,10,11,12,13]. Yet, these approaches are still in their infancy, and despite improved activity against some drug-resistant bacteria, some challenges still remain before clinical use is possible.

Many clinical research reports detail the difficulty in translating *in vitro* combination antibiotic results acquired at the bench into positive patient outcomes [14,15,16]. This is partly attributed to fluctuating pharmacokinetic properties and narrow therapeutic indices [17,18]. Conventional antibiotic combinations have not completely addressed the challenge of treating infections caused by multi-drug-resistant bacteria, especially when incremental and ineffective antibiotic dosing strategies are employed that do not overcome individual mechanisms of resistance [19]. From these studies, it has become apparent that effective therapy not only lies in the judicious choice of agent, but also in the ability to control the delivery and concentration of drugs used in combination [20]. Therefore, effective combination antibiotic therapy is highly dependent on dosing strategies that contain both pharmacophores as one entity, have favorable pharmacokinetics, low toxicity and antimicrobial activity against multi-drug-resistant bacteria.

The literature supports the use of GUMBOS (group of uniform materials based on organic salts) as a chemical approach that could remedy some challenges posed by conventional drug combination therapy [21,22,23,24]. GUMBOS are a new series of hybrid materials composed of at least two functional organic and/or inorganic counter-ions and melt between 25 °C and 250 °C. Previous approaches using low-melting salts, like ionic liquids, have focused more on the desirable physical properties rather than enhancing the active properties and functional uses of the ion pair. However, the limited number of counter-ions that produce nontoxic and functional ionic liquids (Mp <100 °C) makes this approach appear to be a challenging art form instead of a science [25,26,27,28]. Since most ion pairs melt below 250 °C, GUMBOS permit the pairing of any charged pharmaceutical species to obtain tailor-made hydrophobic ion pairs with favorable physical, toxicity, pharmacokinetic and pharmacophoric properties through simple chemical reactions [29,30,31]. GUMBOS represent an avenue that allows charged non-antibiotics and/or antibiotics to be explored inclusively as an adjuvant therapy composed of one molecule, rather than a mixture of unreacted molecules, as done in conventional combination drug therapy [24]. Options for therapy can also be expanded to include outmoded antibiotics, non-antibiotics with membrane altering and potential antibacterial features and mixed-modal systems to facilitate superficial and chronic wound management [27,28,32,33].

**Figure 1:**
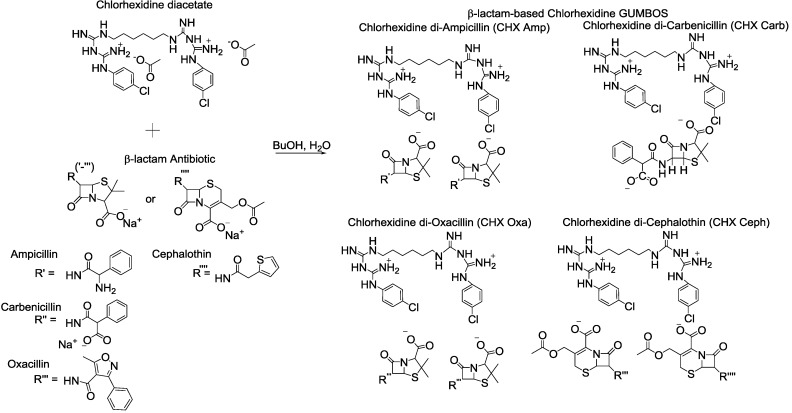
Structures of precursor ions and β-lactam-based chlorhexidine GUMBOS [24].

Herein, the *in vitro* antimicrobial efficacy and synergistic activities of β-lactam-based chlorhexidine GUMBOS (Figure 1) synthesized from outmoded β-lactam antibiotics and the toxic antiseptic, chlorhexidine, against 25 clinical isolates (Table 1) are shown. Comparative analyses between GUMBOS and conventional combination drug studies further confirm the potential of this approach for innovative antimicrobial therapeutic strategies. The toxicities of the GUMBOS were also evaluated with three different cell lines representative of superficial and chronic wound beds.

**Table 1:** Drug-susceptible and drug-resistant bacterial strains.

Strain	Abbreviation	Characteristic
***Escherichia coli 29522 ^+^***	EC 29522	Clinical isolate, quality control organism
***Escherichia coli O157:H7 43895 ^+^***	EC 43895	EHEC, hamburger isolate (*stx*1+, *stx*2+)
***Salmonella typhi ^++^***	Styphi	Fluoroquinolone resistant
***Acinetobacter baumannii 225T2 ^++^***	AB 225T2	Respiratory isolate, multi-drug resistant *
***Acinetobacter baumannii 250 ^++^***	AB 250	Skin isolate, multi-drug resistant
***Acinetobacter baumannii 252 ^++^***	AB 252	Catheter isolate, multi-drug resistant
***Acinetobacter baumannii 254 ^++^***	AB 254	Wound drain isolate, multi-drug resistant
***Enterobacter cloacae 210T2 ^++^***	EC 210T2	Pleural fluid isolate, multi-drug resistant
***Enterobacter aerogenes 221T2 ^++^***	EA 221T2	Sputum, multi-drug resistant
***Klebsiella pneumoniae 10031 ^+^***	KP 10031	Quality control organism
***Klebsiella pneumoniae 50T2 ^++^***	KP 50T2	Urine isolate, multi-drug resistant
***Klebsiella pneumoniae 86T2 ^++^***	KP 86T2	Pleural fluid isolate, multi-drug resistant
***Pseudomonas aeruginosa 124T2 ^++^***	PA 124T2	Respiratory: sputum isolate, β-lactam drug resistant
***Pseudomonas aeruginosa 27853 ^+^***	PA 27853	Blood isolate, quality control organism
***Pseudomonas aeruginosa PSA3 ^++^***	PSA 3	Urine Isolate, β-lactam drug resistant
***Pseudomonas aeruginosa PSA4 ^++^***	PSA 4	Sputum isolate, β-lactam, fluoroquinolone, carbapenem drug resistant
***Serratia marcescens ^++^***	SM	Wound isolate, multi-drug resistant
***Staphylococcus aureus 25923 ^+^***	SA 25923	Clinical isolate
***Streptococcus mutans35668 ^+^***	SM 35668	Quality control organism
***Streptococcus faecalis 19433 ^+^***	SF 19433	Quality control organism
***Micrococcus luteus 4698 ^+^***	ML 4698	Quality control organism
***Streptococcus faecalis 9790 ^+^***	SF 9790	Quality control organism
***Bacillus cereus 1178 ^+^***	BC 1178	Quality control organism
**Methicillin-resistant *Staphylococcus aureus***	CA-MRSA 2	Wound isolate, vancomycin susceptible
**Methicillin-resistan ^++^*CA-MRSA 2*** ***Staphylococcus aureus ^++^ CA-MRSA 1***	CA-MRSA 1	Prosthetic joint infection isolate, vancomycin susceptible

* Multi-drug resistant = β-lactam, fluoroquinolone, carbapenem, aminoglycoside resistant; ^+^ obtained from American Type Culture Collection, Manassas, VA; ^++^ obtained from Jeffrey A. Hobden, Louisiana State University Health Science Center, LA.

## 2. Results and Discussion

### 2.1. Aqueous Solubility, Dissolution and Theoretical Intestinal Absorption

First order dissolution rates were found for GUMBOS after replacing the acetate anion with the β-lactam antibiotic (Table 2). Dissolution rates among the chlorhexidine-based GUMBOS decreased in this order (by antibiotic): oxacillin ≥ ampicillin > cephalothin > carbenicillin; which was found to parallel the order of aqueous solubility of GUMBOS. Reduced solubility as observed for GUMBOS is attributable to greater lipophilicity and increased intermolecular interactions. The reduced aqueous solubility of GUMBOS is directly related to their potential to be more bioavailable. In fact, exchanging the acetate anion with an antibiotic significantly improved estimated intestinal permeability for the GUMBOS (*p* < 0.05). Mean effective permeability coefficients for the GUMBOS were 9.39 × 10^−7^ (±0.87), 4.03 × 10^−6^ (±1.03), 3.67 × 10^−6^ (±0.74), 4.98 × 10^−6^ (±0.087) and 4.91 × 10^−6^ (±0.17) cm/s for chlorhexidine diacetate, chlorhexidine di-ampicillin, chlorhexidine carbenicillin, chlorhexidine di-cephalothin and chlorhexidine di-oxacillin, respectively. Predicted permeability increased four times when chlorhexidine diacetate was converted into a β-lactam-based chlorhexidine GUMBOS. No significant differences were found among permeability coefficients for GUMBOS (*p* < 0.05). This observation suggests that there is a greater chemical difference between the GUMBOS and chlorhexidine diacetate than there is amongst each other, and thus, they may behave as a new ion pair when used therapeutically.

**Table 2:** Summary of aqueous solubility, pharmacokinetic properties and intestinal bioavailability for β-lactam-based chlorhexidine GUMBOS.

Antimicrobial Agent ^a^	Solubility, mg/mL	Dissolution Rate (k), min^−1^	Permeability Coefficients, cm/s (SD)	Log Permeability Coefficients	% HIA ^c^
**CHX Ac**	10	na ^b^	9.39 × 10^−7^ (±0.87)	−6.10	77.4
**CHX Amp**	0.126	0.0188	4.03 × 10^−6^ (±1.03)	−5.39	101.9
**CHX Carb**	0.055	0.0022	3.67 × 10^−6^ (±0.074)	−5.43	100.4
**CHX Ceph**	0.079	0.0037	4.98 × 10^−6^ (±0.082)	−5.30	105.5
**CHX Oxa**	0.166	0.0189	4.91 × 10^−6^ (±0.17)	−5.31	105.3

^a^ CHX Ac: chlorhexidine diacetate; CHX Amp: chlorhexidine di-ampicillin; CHX Ceph: chlorhexidine di-cephalothin; CHX Oxa: chlorhexidine di-oxacillin; ^b^ na: not applicable; ^c^ %HIA: the percent theoretical human intestinal absorption of the drug as determined from the PAMPA permeability assay.

Since simple hydrophobic ion pairing systems have yet to be completely understood, describing the aqueous dissolution of the GUMBOS is not a trivial task. However, what is known to date is that the strength of electrostatic interaction shows little dependence on the size of the ions involved [34]. In general, the dissolution of a salt is not only dependent on differences in stabilities of the ions in water, their hydrophobicity and the energy required to hydrate them, but also on overcoming the energies of other intermolecular forces between these ions. The water-soluble acetate anion in chlorhexidine diacetate has a high charge density and subsequently a large hydration sheath that surrounds each anion. Therefore, less hydration energy is needed to dissolve chlorhexidine diacetate as compared to the anions in the less soluble antibiotic-antiseptic ion pair (e.g., β-lactam-based chlorhexidine GUMBOS). This is because large and/or more hydrophobic ion pairs have stronger electrostatic bonds, despite their asymmetrical lattice and smaller charge densities that limit their interactions with the surrounding aqueous medium and, thus, their aqueous dissociation [34,35]. Lengsfeld *et al.* (2002) classified hydrophobic ion pairs that exhibited such behavior as units that form intermediate tight or loose pairs rather than salts that completely dissolve into individual ions [32,34,35]. This is especially evident for the GUMBOS when comparing two structurally similar anions (*i.e*., ampicillin and carbenicillin), where it was found that chlorhexidine di-ampicillin (symmetrical, two anions) behaved more as a loose ion pair with a higher dissolution rate (~9×) and greater aqueous solubility (~2×) than chlorhexidine carbenicillin (asymmetrical, one anion and tight pair). The marked differences in solubility and intestinal permeability values between chlorhexidine diacetate and GUMBOS support the contention that the GUMBOS ions are travelling as one molecule and not as mixed, separate ions. This outcome is further supported by the antibacterial results.

### 2.2. Antibacterial Activities of β-Lactam-Based Chlorhexidine GUMBOS

The antibacterial activity of the precursor components to the GUMBOS were assessed against each parent ion individually and as a stoichiometrically equivalent mixture (Table 3, Table 4 and Table 5). Initial antibacterial studies with β-lactam antibiotics and chlorhexidine diacetate were found most effective against drug-susceptible bacteria, in which the β-lactam antibiotics were preferential toward Gram-positive bacteria (Gram-positive bacteria) and within acceptable MIC ranges and chlorhexidine diacetate against Gram-negative bacteria (GNB) (Table 3 and Table 4). As expected, extremely high concentrations of β-lactam antibiotic were required to inhibit the growth of the multi-drug-resistant isolates and required concentrations greater than 1250 µM for growth inhibition.

**Table 3:** MICs (μM) of β-lactam antibiotics, chlorhexidine diacetate and four GUMBOS against 8 clinical isolates of Gram-positive bacteria with β-lactam antibiotic resistance ^a^.

Antibacterial Agent ^b^									
Gram-Positive Bacteria	Amp	Carb	Ceph	Oxa	CHX Ac	CHX Amp	CHX Carb	CHX Ceph	CHX Oxa
SA 25923	2	13	125	125	1	0.9	0.5	0.4	0.5
SM 35668	0.8	94	104	98	0.4	0.4	0.4	0.4	0.4
SF 19433	0.2	0.1	0.1	0.1	0.8	0.4	0.8	0.8	0.4
ML 4698	625	>1250	>1250	>1250	0.8	0.6	0.5	0.3	0.1
SF 9790	625	>1250	>1250	>1250	0.8	0.8	0.8	0.8	0.8
BC 1178	625	>1250	>1250	>1250	0.4	0.4	0.4	0.4	0.2
CA-MRSA 2	625	>1250	>1250	>1250	1	0.8	0.7	0.4	0.2
CA-MRSA 1	>1250	>1250	>1250	>1250	0.7	0.7	0.3	0.3	0.2

^a^ The maximum concentration tested was 1250 μM; ^b^ Amp: sodium ampicillin; Carb: carbenicillin disodium, Ceph: sodium cephalothin; Oxa: sodium oxacillin; CHX Ac: chlorhexidine diacetate; CHX Amp: chlorhexidine di-ampicillin; CHX Carb: chlorhexidine carbenicillin; CHX Ceph: chlorhexidine di-cephalothin; CHX Oxa: chlorhexidine di-oxacillin.

**Table 4:** MICs (μM) of β-lactam antibiotics, chlorhexidine diacetate and four GUMBOS against 17 clinical isolates of Gram-negative bacteria of varying antibiotic resistance ^a^.

Antibacterial Agent ^b^									
Gram-Negative Bacteria	Amp	Carb	Ceph	Oxa	CHX Ac	CHX Amp	CHX Carb	CHX Ceph	CHX Oxa
EC 29522	>1250	>1250	>1250	>1250	0.3	0.3	0.2	0.2	0.2
EC 43895	>1250	>1250	>1250	>1250	0.2	0.1	0.1	0.1	0.1
Styphi	>1250	>1250	>1250	>1250	0.2	0.2	0.1	0.1	0.1
AB 225T2	>1250	>1250	>1250	>1250	12	12	15	20	15
AB 250	>1250	>1250	>1250	>1250	20	24	7	7	7
AB 252	>1250	>1250	>1250	>1250	29	20	20	20	20
AB 254	>1250	>1250	>1250	>1250	4	4	10	7	10
EC 210T2	>1250	>1250	>1250	>1250	15	20	24	24	10
EA 221T2	>1250	>1250	>1250	>1250	16	24	20	20	10
KP 10031	27	22	29	17	5	13	13	13	13
KP 50T2	540	270	270	215	5	12	20	20	10
KP 86T2	14	28	28	28	6	7	10	20	10
PA 124T2	10	10	10	5	22	10	15	10	15
PA 27853	28	28	28	28	6	8	8	3	3
PSA 3	32	39	27	45	4	12	15	10	10
PSA 4	>1250	>1250	>1250	>1250	6	20	20	15	10
SM	>1250	>1250	>1250	>1250	9	22	22	32	16

^a^ The maximum concentration tested was 1250 μM; ^b^ Amp: sodium ampicillin; Carb: carbenicillin disodium, Ceph: sodium cephalothin; Oxa: sodium oxacillin; CHX Ac: chlorhexidine diacetate; CHX Amp: chlorhexidine di-ampicillin; CHX Carb: chlorhexidine carbenicillin; CHX Ceph: chlorhexidine di-cephalothin; CHX Oxa: chlorhexidine di-oxacillin.

**Table 5:** MICs (μM) and FICI of four GUMBOS and their unreacted mixtures at stoichiometric equivalence against three quality control strains ^a^.

	CHX Amp	1 CHX:2 Amp	CHX Carb	1 CHX:1 Carb	CHX Ceph	1 CHX:2 Ceph	CHX Oxa	1 CHX:2 Oxa
SA 25923								
MIC	0.9	2.4	0.5	18.8	0.4	9.4	0.5	2.0
FICI ^b^	0.3	0.2	0.3	7.1	0.1	3.4	0.2	1.3
Effect	syn	syn	syn	ant	syn	add	syn	add
KP 10031								
MIC	12.5	37.5	12.5	37.5	12.5	18.8	12.5	18.8
FICI	0.9	2.7	1.3	2.7	0.9	1.5	0.9	2.0
Effect	add	add	add	add	add	add	add	add
PA 27853								
MIC	7.8	18.8	7.8	18.8	3.1	9.4	3.1	18.8
FICI	0.4	1.3	0.6	1.5	0.2	0.8	0.2	1.5
Effect	syn	add	add	add	syn	add	syn	add

^a^ 1 CHX: 2 Amp: 1 mole chlorhexidine diacetate: 2 moles sodium ampicillin; CHX Amp: chlorhexidine di-ampicillin; 1 CHX: 1 Carb: 1 mole chlorhexidine diacetate: 1 mole disodium carbenicillin; CHX Carb: chlorhexidine carbenicillin; 1 CHX: 2 Ceph: 1 mole chlorhexidine diacetate: 2 moles sodium cephalothin; CHX Ceph: chlorhexidine di-cephalothin; 1 CHX: 2 Oxa: 1 mole chlorhexidine diacetate: 2 moles sodium oxacillin; CHX Oxa: chlorhexidine di-oxacillin; ^b^ Fractional Inhibitory Concentration Interaction Index (FICI) ≤ 0.5, synergy (syn); 0.5 < FICI ≤ 3, additivity (add); FICI > 3, antagonism (ant).

Antibacterial activity against Gram-positive bacteria, including *MRSA*, are listed in Table 3. GUMBOS inhibited drug-susceptible Gram-positive bacteria including *MRSA* with <0.7 µM (0.56 µg/mL). Lower concentrations of GUMBOS were required to inhibit the growth of Gram-positive bacteria and *MRSA* than that found for chlorhexidine diacetate. On average, GUMBOS required approximately half the moles than chlorhexidine diacetate to inhibit Gram-positive bacteria. However, GUMBOS needed 25, 40, 2444, and 3400× fewer moles than β-lactam antibiotics to inhibit *Micrococcus luteus* (*ML*) 4698, *SA* 25,923 and both *MRSA* isolates, CA-MRSA 2 and CA-MRSA 1, respectively. Consequently, antibacterial activity was improved in the chlorhexidine ion pair after the acetate was exchanged for the antibiotic, which increased similarly with known antibiotic efficacy against Gram-positive bacteria: acetate < ampicillin ≤ carbenicillin < cephalothin < oxacillin. Comparable results for Gram-positive bacteria, including *MRSA*, to the chlorhexidine ion-pairs suggest that synergy may exist between the chlorhexidine and antibiotics when used as GUMBOS. This result is in spite of the β-lactam resistance of the *MRSA* strains. Thus, GUMBOS’s superior activity is attributable to the bulky nature of the chlorhexidine cation that may have sterically hindered β-lactamase attachment and/or deactivation of the antibiotic pharmacophore in the *MRSA* strains. Future studies are underway to evaluate β-lactamase activities in the presence of these GUMBOS, as this method may impart a new treatment approach for resistant infections in which enzymes are used in antibiotic deactivation mechanisms.

Antibacterial activities upon GNB are illustrated in Table 4. GUMBOS concentrations to inhibit drug-susceptible GNB followed a similar trend as that observed with Gram-positive bacteria and required nearly half the concentration of chlorhexidine diacetate. Significant improvements (*i.e*., 300–710×) in antibacterial activity were mostly seen for *EC* 29522 and *EC* 43895 isolates when comparing β-lactam antibiotics and GUMBOS (*p* < 0.05). These findings agree with previous reports by Cole *et al*. (2013) [24]. There was no significant difference in mean antibacterial activities between β-lactams and GUMBOS against *S. typhi* (*p* < 0.05).

Antibacterial activity was species-dependent for the multi-drug-resistant Gram-negative bacteria tested (Table 4). Overall, GUMBOS inhibited the growth of multi-drug-resistant Gram-negative bacteria in this order: *S. marcescens < K. pneumoniae < E. cloacae < P. aeruginosa < A. baumannii*; in which the least antibacterial activity was observed on *S. marcescens.* In comparing the individual activity of each GUMBOS, it was found that specific β-lactam-based chlorhexidine ion pairs (GUMBOS) were more effective towards certain genera of multi-drug-resistant Gram-negative bacteria. For instance, chlorhexidine di-ampicillin outperformed other GUMBOS when inhibiting *A. baumannii*. Comparable inhibition of *P. aeruginosa* was observed for all GUMBOS and chlorhexidine diacetate. Chlorhexidine di-oxacillin was twice more effective towards *E. cloacae* than the other GUMBOS and chlorhexidine diacetate. GUMBOS preferentially inhibited neither *K. pneumoniae* nor *S. marcescens* and required a 2–4× greater concentration than chlorhexidine diacetate for similar activity. Exchanging acetate in chlorhexidine salts for a β-lactam antibiotic yielded superior antibacterial activity against 57% of multi-drug-resistant Gram-negative bacteria isolates evaluated in this study. Mean concentrations required to inhibit drug-susceptible GNB and multi-drug-resistant Gram-negative bacteria were 0.1 ± 0.06 µM and 14 ± 6 µM, respectively. Overall, GUMBOS inhibited 88% of the multi-drug-resistant Gram-negative bacteria tested with less than 20 µM (23.5 µg/mL), and 45% of that population was inhibited under 10 µM (11.7 µg/mL).

Examination of several literature reports suggests that extraneous anions impair the antibacterial activity of chlorhexidine salts [36,37]. Since such issues can limit some combination drug studies and the use of outmoded antibiotics (or other previously approved non-antibiotics) in the form of GUMBOS, the combined interactions of chlorhexidine diacetate and various β-lactam antibiotics at appropriate, equal stoichiometry were evaluated to determine if this applies to GUMBOS or if it is exclusive to their unreacted mixtures. The antibacterial efficacy of GUMBOS and unreacted mixtures against *S. aureus* 29523, *K. pneumoniae* 10031 and *P. aeruginosa* 27853 are listed in Table 5.

### 2.3. Comparison of Antibacterial Activity between Reacted β-Lactam-Based Chlorhexidine GUMBOS and Its Unreacted Mixtures

Table 5 lists the antibacterial activity of the reacted GUMBOS and unreacted mixture of chlorhexidine diacetate and β-lactam antibiotic. Mixtures of chlorhexidine diacetate and β-lactam antibiotic were most effective on *S. aureus* 29523 and increased (by antibiotic) in this order: ampicillin ≅ carbenicillin (2 μM) < cephalothin (9 μM) < oxacillin (19 μM). This trend is opposite to the strength of the antibiotics when used alone and at 3–11× less concentration (*p* < 0.05). However, neither mixture used fewer moles to inhibit *S. aureus* 29523 than chlorhexidine diacetate nor was more effective than their respective GUMBOS. Interaction indices determined by MIC values against *S. aureus* 29523 support that synergy (Fractional Inhibitory Concentration Interaction Index, FICI <0.5) occurred exclusively as a GUMBOS rather than the combined, unreacted mixture of chlorhexidine diacetate and sodium antibiotic. Higher interaction indices (FICI >3) suggest that antagonism was evident when chlorhexidine diacetate was mixed with β-lactam antibiotics, although both individually were effective in inhibiting *S. aureus* 29523. Regardless of being the most effective antibiotic towards *S. aureus* 29523, antagonism was greatest when sodium oxacillin was mixed with chlorhexidine diacetate. Synergetic mixtures with less antagonism decreased with increasing β-lactam strength, hydrophobicity and size. This result corroborates previous findings regarding extraneous ions interfering with the antibacterial activity of chlorhexidine salts. Thus, GUMBOS are truly interacting with bacteria as one entity and not as two separate antimicrobials.

Since *K. pneumoniae* 10031 and *P. aeruginosa* 27853 are clinically resistant to many antibiotics, including the β-lactam class, it was expected that worse antibacterial activity would be observed as compared to that found for *S. aureus* 29523. In each instance, the GUMBOS were more effective than the unreacted mixture of precursor ions. For these microrganisms, the more hydrophobic antibiotics (*i.e*., sodium cephalothin and sodium oxacillin) combined with chlorhexidine diacetate resulted in better FICI values than that observed with *S. aureus* 29523. All interactions between chlorhexidine diacetate and sodium antibiotic were additive when inhibiting *K. pneumoniae* 10031. Likewise, GUMBOS were found to have an additive interaction index (0.5 < FICI < 3). Thus, neither chlorhexidine diacetate nor β-lactam antibiotic potentiated the antibacterial activity of the other against this microorganism as either a mixture or as a reacted ion pair. Additivity was also found for *P. aeruginosa* 27853 when a stoichiometric equivalent mixture of chlorhexidine diacetate and sodium antibiotic were used. Yet, the greatest instance of synergy was found when chlorhexidine di-oxacillin was used; whereas, interactions shifted towards additivity as less hydrophobic and smaller antibiotics were used in the antimicrobial mixture against *P. aeruginosa* 27853.

**Figure 2:**
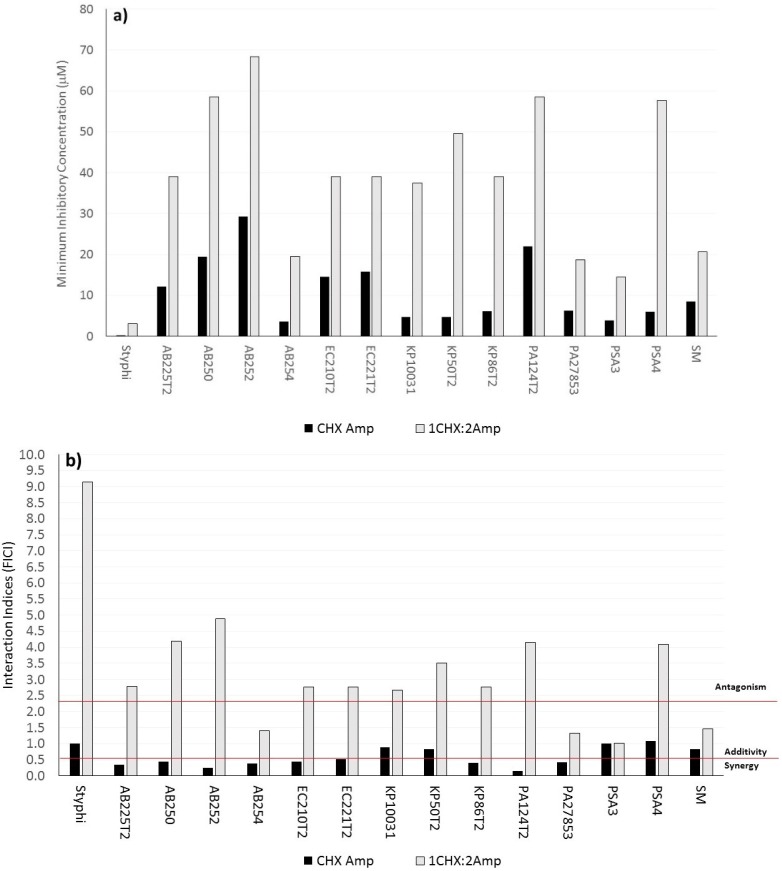
(**a**) MICs and (**b**) FICIs of chlorhexidine di-ampicillin (CHX Amp) GUMBOS and the unreacted stoichiometric equivalent (1 CHX: 2 Amp mixture) against 15 multi-drug-resistant Gram-negative bacteria.

**Figure 3:**
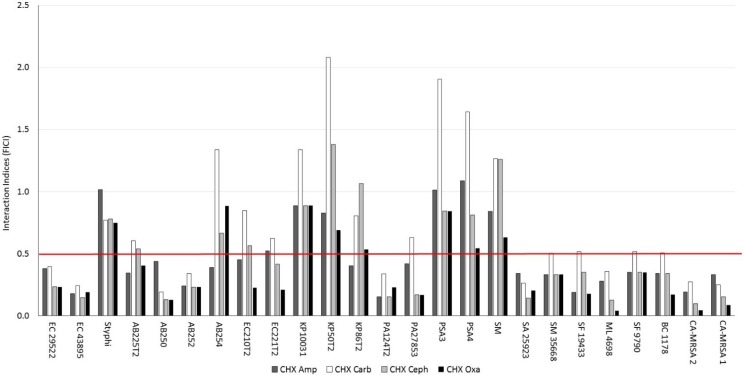
FICIs of β-lactam-based chlorhexidine GUMBOS determined on 17 clinical isolates of Gram-negative bacteria of varying antibiotic resistance.

A similar response was found upon multi-drug-resistant Gram-negative bacteria treated with chlorhexidine di-ampicillin GUMBOS (Figure 2a). In 92% of the isolates tested, reduced MICs were needed to inhibit multi-drug-resistant Gram-negative bacteria growth. Approximately 15 and 37 μM of chlorhexidine di-ampicillin and its stoichiometric equivalent mixture of chlorhexidine diacetate and sodium ampicillin, respectively, were needed to inhibit multi-drug-resistant Gram-negative bacteria. This approximate two-fold difference in MIC values between the unreacted mixture and chlorhexidine di-ampicillin was apparent against all multi-drug-resistant Gram-negative bacteria, except *S. marcescens*. Most of the unreacted mixtures of chlorhexidine diacetate and sodium ampicillin were additive, although there were few instances of antagonism among isolates (Figure 2b). Synergy was determined for chlorhexidine di-ampicillin GUMBOS among 69% of the multi-drug resistant Gram-negative bacteria isolates (Figure 2b). Figure 3 summarizes the interaction indices for GUMBOS against Gram-positive bacteria and GNB revealing that synergy was evident for most of the microorganisms tested.

### 2.4. Cytotoxicity and Therapeutic Indices of Chlorhexidine and β-Lactam Antibiotics in Combination and as GUMBOS

Adjunctive wound treatment options are successful in removing the source of infection, but can exacerbate damage to the surrounding tissues and interrupt the natural wound healing process if too potent and consequently toxic. This issue is a critical area in which many effective antimicrobial drug therapies fail [3,38,39]. It is known that β-lactam antibiotics are highly nontoxic (>500 µM); however, issues of toxicity caused by systemic administration of chlorhexidine diacetate limit its therapeutic application. In order for GUMBOS to be a feasible alternative to combination drug treatment strategies, concerns for chlorhexidine toxicity, despite its approved topical use, needs to be minimized. Thus, cytotoxicity and therapeutic indices of the GUMBOS and their stoichiometric mixtures upon mammalian cells critical to superficial and chronic wounds were evaluated to realize the utility of GUMBOS in wound treatment regimens initiated by multi-drug-resistant Gram-negative bacteria (Table 6).

**Figure 4:**
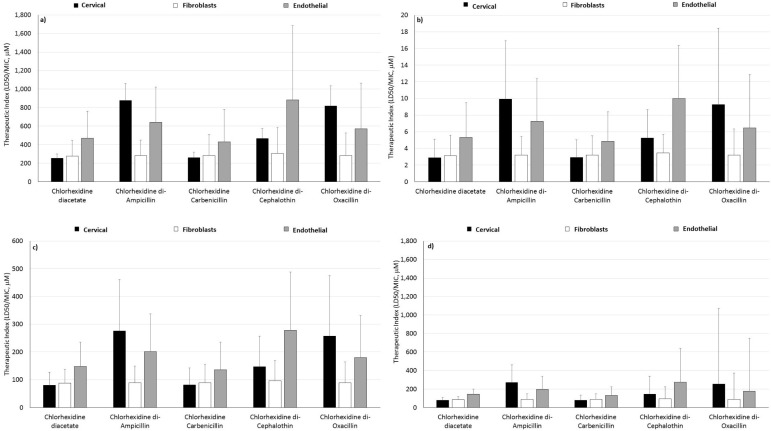
Theoretical therapeutic indices of β-lactam-based chlorhexidine GUMBOS determined from (**a**) drug-susceptible Gram-negative bacteria, (**b**) multi-drug-resistant Gram-negative bacteria, (**c**) drug-susceptible Gram-positive bacteria and (**d**) *MRSA*, against cervical, fibroblasts and endothelial cell lines.

Fibroblasts were chosen to simulate the cell type responsible for healing superficial wounds or abrasions. Chlorhexidine diacetate is a common antiseptic used to manage superficial abrasions. As expected, chlorhexidine diacetate and the β-lactam antibiotics were equally nontoxic to fibroblast cells. Likewise, the stoichiometric mixture and GUMBOS were equally nontoxic to the fibroblast cells as the parent drugs. These results suggest that GUMBOS can also be used as topical disinfectants without interfering with the proliferative phase of the wound healing process that occurs during superficial or chronic wound recovery. Therapeutic indices for each chlorhexidine GUMBOS and fibroblasts cells decreased in this order: drug-susceptible Gram-negative bacteria > drug-susceptible Gram-positive bacteria = *MRSA* > multi-drug resistant Gram-negative bacteria (Figure 4). Variation among the therapeutic indices determined for the GUMBOS were statistically insignificant (*p* < 0.05).

**Table 6:** Acute cytotoxicity (LD_50_, µM) of β-lactam-based chlorhexidine GUMBOS.

Antibacterial Agents ^a^	Cervical	Fibroblasts	Endothelial
**CHX Ac**	43 ± 3	47 ± 2	80 ± 3
**1 CHX:2 Amp**	76 ± 4	43 ± 2	67 ± 11
**CHX Amp**	149 ± 8	48 ± 3	109 ± 6
**1 CHX:1 Carb**	58 ± 3	51 ± 4	59 ± 4
**CHX Carb**	44 ± 2	48 ± 7	73 ± 10
**1 CHX:2 Ceph**	65 ± 6	52 ± 6	103 ± 14
**CHX Ceph**	79 ± 2	52 ± 5	150 ± 13
**1 CHX:2 Oxa**	92 ± 3	44 ± 4	92 ± 7
**CHX Oxa**	139 ± 8	48 ± 4	97 ± 16

^a^ 1 CHX: 2 Amp: 1 mole chlorhexidine diacetate: 2 moles sodium ampicillin; CHX Amp: chlorhexidine di-ampicillin; 1 CHX: 1 Carb: 1 mole chlorhexidine diacetate: 1 mole disodium carbenicillin; CHX Carb: chlorhexidine carbenicillin; 1 CHX: 2 Ceph: 1 mole chlorhexidine diacetate: 2 moles sodium cephalothin; CHX Ceph: chlorhexidine di-cephalothin; 1 CHX: 2 Oxa: 1 mole chlorhexidine diacetate: 2 moles sodium oxacillin; CHX Oxa: chlorhexidine di-oxacillin.

Chronic wounds, like diabetic foot ulcers, severe burns or non-healing surgical wounds, often provide an optimal climate for opportunistic drug-resistant infections to persist and result in blood sepsis [3,38]. Since the vascularity of chronic wounds is limited, it is difficult to administer effective systemic therapy for these ailments. Moreover, potent antimicrobials may aggravate the thin endothelial lining and interfere with the wound healing process during treatment. Therefore, *in vitro* cytotoxicity of GUMBOS and the unreacted drug pair upon endothelial cells was evaluated to determine if GUMBOS were an effective, nontoxic treatment for chronic wounds caused by multi-drug-resistant bacteria. Our findings show that unreacted drug mixtures containing stoichiometric equivalents of chlorhexidine diacetate and sodium antibiotic were more cytotoxic than the respective GUMBOS (Table 6). Overall, endothelial cytotoxicity of the investigated GUMBOS and corresponding stoichiometric drug combinations, from least to greatest, occurred in this order (by anion): cephalothin < ampicillin < oxacillin < carbenicillin. In comparison to fibroblasts, nearly 2–3× greater concentrations of GUMBOS could be used to manage chronic wounds. As the status of the wound progresses from chronic to superficial, however, the concentration of GUMBOS would need to be reduced. Therapeutic indices for each chlorhexidine GUMBOS with endothelial cells were the same as fibroblasts, and theoretical dosing options were at least twice as flexible (Figure 4). Variations among therapeutic indices were statistically significant (*p* < 0.05) and were reflected among each group of bacteria studied.

For a general approximation of systemic cytotoxicity and probable use in eradicating infections of the cervix, cervical cells were used. It was found that cervical toxicity caused by GUMBOS increased in this order (by anion): ampicillin < oxacillin < cephalothin ≤ carbenicillin. Chlorhexidine carbenicillin showed indifferent toxicity to chlorhexidine diacetate, while the other GUMBOS and mixtures were less toxic to cervical cells (Table 6). In comparison to chlorhexidine diacetate, therapeutic indices for GUMBOS improved as much as five-times as compared to chlorhexidine diacetate (Figure 4). Broad therapeutic indices were also found for GUMBOS and cervical cells in an order similar to the other cell lines. The greatest range of therapy was found for chlorhexidine di-ampicillin and chlorhexidine di-oxacillin. Variation among therapeutic indices was statistically significant (*p* < 0.05).

### 2.5. Limitations

These results also demonstrate that this approach may have its limitations, as well. There is a possibility that each drug combination may not show any difference in activity or toxicity as compared to the precursor drug or its use in an unreacted mixture. An example of this is found when comparing the structurally similar chlorhexidine carbenicillin to chlorhexidine di-ampicillin. Throughout this study, we have observed superior performance of chlorhexidine di-ampicillin to that of chlorhexidine carbenicillin. In fact, chlorhexidine carbenicillin typically was equally cytotoxic as chlorhexidine diacetate. More specifically, chlorhexidine carbenicillin was 2–4× more cytotoxic than chlorhexidine di-ampicillin. This is attributable to the difference in structure and resultant stoichiometry between the cation and anion constituents. Carbenicillin and ampicillin are structurally similar with the exception of the carboxylate or primary amine, respectively located on the C-8 position of the antibiotic. This secondary carboxylate on carbenicillin allows it to be di-anionic as compared to the mono-anionic charge of ampicillin. After reacting the chlorhexidine with either antibiotic, a 1:1 chlorhexidine-carbenicillin stoichiometry and 1:2 chlorhexidine-ampicillin stoichiometry are achieved. Although experiments are still underway to understand the role of stoichiometry in the antibacterial and cytotoxic properties of GUMBOS, we believe that the structural configuration of the 1:2 stoichiometric GUMBOS as compared to the 1:1 stoichiometric GUMBOS affects the properties reported herein. This is especially since all 1:2 chlorhexidine-β-lactam antibiotic GUMBOS were continuously superior to chlorhexidine carbenicillin.

## 3. Experimental Section

### 3.1. Antibacterial Agents

Chlorhexidine diacetate, sodium ampicillin, sodium oxacillin, sodium cephalothin, disodium carbenicillin, methanol, dimethylsulfoxide, 3-(4,5-dimethyl-2-thiazyl)-2,5-diphenyl-2H-tetrazolium bromide and Corning 3695 flat-well 96-well plates were purchased from Sigma Aldrich (St. Louis, MO, USA) and used without further purification. Cation-adjusted Mueller–Hinton broth and the BD Gentest Pre-coated PAMPA Plate System were obtained from BD Biosciences (Franklin Lakes, NJ, USA).

### 3.2. Synthesis of β-Lactam-Based Chlorhexidine GUMBOS

The synthesis and physical characterization of four β-lactam-based chlorhexidine GUMBOS, namely chlorhexidine di-ampicillin, chlorhexidine carbenicillin, chlorhexidine di-cephalothin and chlorhexidine di-oxacillin, were performed using methods previously reported by Cole *et al.* (2013) [24], but with slight modification. Briefly, stoichiometric amounts of chlorhexidine diacetate and β-lactam antibiotic, with the latter in slight excess, was stirred for 48 h at room temperature in a butanol:water (1:1) mixture to ensure the complete formation of the β-lactam-based chlorhexidine GUMBOS. After removing butanol from the GUMBOS products, they were purified by washing several times with cold deionized water and dried overnight with a high vacuum. The structures of chlorhexidine di-ampicillin, chlorhexidine carbenicillin, chlorhexidine di-oxacillin and chlorhexidine di-cephalothin (Figure 1) were mainly confirmed by NMR, mass spectrometry and elemental analysis, among other spectroscopic data.

*Chlorhexidine di-ampicillin*. Off-White Solid, yield 98%, Water solubility: 126 μg/mL. Solubility product constant (Ksp): 4.63 × 10^−12^ M^3^. ^1^H-NMR (400 Hz, DMSO-*d_6_*) δ 8.58–8.36 (m, 2 H) 7.21–7.50 (m, 18 H), 5.08 (d, *J =* 2.74 Hz, 2 H), 4.96 (s, 4 H), 3.69 (d, *J =* 3.13 Hz, 2 H), 3.26 (s, 2 H), 3.07 (dt, *J =* 7.04, 6.65 Hz, 4 H), 1.85 (s, 4 H), 1.57 (s, 6 H), 1.49 (s, 4 H), 1.46 (quin, 4 H), 1.44 (s, 4 H), 1.27 (quin, 4 H), 1.17 (s, 6 H), 1.15 (s, 2 H). ^13^C-NMR (101 MHz, DMSO) δ 180.88, 72.88, 172.36, 166.94, 166.76, 139.07, 128.24, 127.01–128.43, 121.90, 76.07, 68.38, 60.80, 60.01, 58.64, 27.38, 26.76, 25.97. Anal. calcd. for C_54_H_68_Cl_2_N_16_O_8_S_2_: C, 53.86; H, 5.69; Cl, 5.89; N, 18.61; O, 10.63; S, 5.33. Found: C, 53.22; H, 5.81; Cl, 5.56; N, 18.37; S, 5.16. HRMS (ESI) *m/z* calcd. for C_54_H_68_Cl_2_N_16_O_8_S_2_, [M+H^+^], 1203.4424; found, 1203.4136.

*Chlorhexidine carbenicillin*. Colorless Solid, yield 93%, Water solubility: 55 μg/mL. Ksp: 3.53 × 10^−9^ M^2^. ^1^H-NMR (400 Hz, DMSO-*d_6_*) δ 0.69–0.76 (m, 1 H) 0.96 (s, 1 H) 1.06 (s, 2 H) 1.15 (s, 4 H) 1.34 (s, 4 H) 1.40–1.54 (m, 5 H) 1.61 (s, 2 H) 2.96 (s, 4 H) 3.07 (s, 1 H) 3.16–3.34 (m, 3 H) 3.43 (d, *J =* 5.14 Hz, 1 H) 3.47–3.51 (m, 1 H) 3.52–3.56 (m, 1 H) 3.92–1303.99 (m, 1 H) 4.18–4.27 (m, 1 H) 4.82 (s, 1 H) 6.96–7.20 (m, 9 H) 7.28 (s, 3 H) 7.36–7.68 (m, 2 H) 8.24–8.35 (m, 1 H). ^13^C-NMR (101 MHz, DMSO) δ 128.71, 122.65, 108.20, 40.40, 40.19, 26.42. Anal. calcd. for C_39_H_48_Cl_2_N_12_O_6_S: C, 53.00; H, 5.47; Cl, 8.02; N, 19.02; O, 10.86; S, 3.63. Found: C, 51.12; H, 5.68; Cl, 7.74; N, 18.34; S, 3.50. HRMS (ESI) *m/z* calcd. for C_39_H_48_C_l2_N_12_O_6_S, [M+], 883.8462; found, 883.8457.

*Chlorhexidine di-cephalothin.* Orange Solid, yield 83%, Water solubility: 79 μg/mL. Ksp: 1.89 × 10^−11^ M^3^. ^1^H-NMR (400 MHz, DMSO-*d_6_*) δ1.27 (s, 5 H) 1.45 (s, 6 H) 1.79 (d, *J =* 3.42 Hz, 1 H) 2.01 (s, 6 H) 3.07 (s, 5 H) 3.17 (s, 3 H) 3.27(d, *J =* 17.61 Hz, 3 H) 3.33 (s, 4 H) 3.50 (d, *J =* 17.36 Hz, 3 H) 3.77 (d, *J =* 2.93 Hz, 4 H) 4.79 (d, *J =* 11.98 Hz, 2 H) 5.00 (d, *J =* 4.65 Hz, 3 H) 5.03(s, 1 H) 5.53 (dd, *J =* 8.31, 4.89 Hz, 2 H) 6.89–6.98 (m, 5 H) 7.29 (d, *J =* 8.80 Hz, 6 H) 7.33–7.38 (m, 2 H) 7.44 (d, *J =* 8.31 Hz, 13 H) 9.03 (d, *J =* 8.31 Hz, 2 H).^13^C-NMR (101 MHz, DMSO) δ 170.97, 170.41, 165.77, 163.59, 137.47, 134.44, 128.71, 127.04, 126.69, 125.39, 122.47, 113.60, 64.77, 59.12, 57.67, 40.63, 40.42, 36.23, 26.42, 25.64, 21.17. Anal. calcd. for C_54_H_62_Cl_2_N_14_O_12_S_4_: C, 49.96; H, 4.81; Cl, 5.46; N, 15.10; O, 14.79; S, 9.88. Found: C, 48.61; H, 4.99; Cl, 5.31; N, 14.70; S, 9.61. HRMS (ESI) *m/z* calcd. for C_54_H_62_Cl_2_N_14_O_12_S_4_, [M+], 1298.3227; found, 1298.3199.

*Chlorhexidine di-oxacillin.* Colorless Solid, yield 85%, Water solubility: 166 μg/mL. Ksp: 8.38 × 10^−12^ M^3^. ^1^H-NMR (400 MHz, DMSO-*d_6_*) δ 8.99 (d, *J* = 9.0 Hz, 4H), 7.90–7.85 (m, 2H), 7.76 (d, *J =* 7.6 Hz, 4H), 7.71–7.64 (s, 1H), 7.60–7.52 (m, 2 H). 7.52–7.43 (m, 15H), 7.38 (t, *J* = 7.4 Hz, 2H), 7.28 (d, *J* = 8.4Hz), 4.96 (d, *J =* 8.0 Hz, 2 H), 4.61 (t, *J* = 8.4 Hz, 2H), 3.67 (s, 8 H), 3.40 ( s., 1 H), 3.27 (s, 2H), 3.05 (s, 4 H), 1.55 (s, 6H), 1.43 (m, 4H), 1.25(m, 4H), 1311.19 (s, 6H). ^13^C-NMR (101 MHz, DMSO) δ 173.07, 1708.51, 169.46, 161.59, 159.98, 138.94, 129.85, 128.77, 128.22, 127.87, 121.84, 112.43, 75.22, 65.84, 59.67, 57.77, 52.01, 27.83, 26.02, 11.81. Anal. calcd. for C_60_H_68_C l_2_N_16_O_10_S_2_: C, 55.08; H, 5.24; Cl, 5.42; N, 17.13; O, 12.23; S, 4.90. Found: C, 53.61; H, 5.40; Cl, 5.27; N, 16.67; S, 4.77. HRMS (ESI) *m/z* calcd. for C_60_H_68_Cl_2_N_16_O_10_S_2_, [M+], 1308.3191; found, 1308.30.

### 3.3. Bacterial Strains

Twenty-five clinical isolates were obtained from critically ill patients sent to Professor Jeffrey A. Hobden, Louisiana State University Health Science Center, LA, for drug-resistance screening and antibiotic susceptibility.

### 3.4. Media

Cation-adjusted Mueller–Hinton broth (MHB, Becton Dickinson and Company, Franklin Lakes, NJ, USA) with 1% DMSO was used to determine minimum inhibitory concentrations (MICs) and synergy experiments.

### 3.5. Minimum Inhibitory Concentrations and Synergy Experiments

MICs of GUMBOS, parent ions and unreacted mixtures were determined by standard broth dilution in 96-well microtiter plates. GUMBOS, parent ions and the unreacted mixtures (500 mM) were dissolved in DMSO to prepare a stock solution of known concentration. Then, 1 µL of each stock was injected into wells containing 100 µL MHB to a starting concentration of 5000 µM (1% DMSO). The compounds were serially diluted (1:1) to yield a concentration range between 0.1 and 2500 μM at a 100-μL volume. Equal volume (100 μL) of starting inocula matching a 0.5 McFarland standard were added to each well and incubated at 37 °C for 24 h. Unreacted mixtures consisted of stoichiometric equivalents of chlorhexidine and sodium antibiotic to the GUMBOS (*i.e*., 1 mole of chlorhexidine diacetate and 2 moles of ampicillin, cephalothin and oxacillin or 1 mole of carbenicillin). Turbidity was used as an initial indication of microbial growth, and if present, the corresponding concentration of antibacterial agent was considered ineffective. Absorbance was recorded 30 minutes after injecting 20 µL MTT (1 mg/mL) and 80 μL lysing solution (30% sodium dodecyl sulfate and 10% DMSO) in to each well to determine percent inhibition values for each concentration tested per organism. To eliminate differences in concentrations that arise from molecular weight differences of GUMBOS, precursor ions and their unreacted mixtures, molar concentrations were used to compare the antibacterial activities and for statistical analysis. This would result in micromolar MIC values that are not of conventional dilution concentrations. Antibacterial activity was analyzed by analysis of variance (ANOVA) with comparisons of means to check statistical significance using SAS 9.2 2 (SAS Institute Inc., Cary, NC, USA), *p* < 0.05.

### 3.6. Interaction Indices and Synergy Testing

Loewe’s additivity model was used to assess the fractional interaction index (FICI) between ion pairs for the reacted GUMBOS and unreacted ion mixture (chlorhexidine diacetate + sodium antibiotic) [24]. Minimum inhibitory concentrations acquired for stoichiometric mixtures and GUMBOS were used to tabulate FICI values. The FICI values were interpreted as follows: (1) FICI ≤ 0.5, synergy; (2) 0.5 ≤ FICI ≤ 3, additive or neutral; and (3) FICI >3, antagonism. Equations (1)–(3) were used to calculate FICI values between the ions in the GUMBOS or the unreacted, stoichiometric mixture of ions. Chlorhexidine di-ampicillin, chlorhexidine di-cephalothin and chlorhexidine di-oxacillin were determined with Equation (2) to calculate FICI values; whereas, chlorhexidine carbenicillin with Equation (3).

(1) $${FICI}_{\text{combo}}=\frac{{\left[CHX\right]}_{COMBO}}{{\left[CHXAc\right]}_{100\%}}+\frac{{\left[\beta -lactam\right]}_{COMBO}}{{\left[\beta -lactam\right]}_{100\%}}$$

(2) $${FICI}_{GUMBOS}=\frac{0.33\times [CHXAmp_{2}]}{[CHX]{\left[Ac_{2}\right]}_{100\%}}+\frac{0.66\times [CHXAmp_{2}]}{[Na]{\left[Amp\right]}_{100\%}}$$

(3) $${FICI}_{GUMBOS}=\frac{0.50\times \left[CHXCarb\right]}{[CHX]{\left[Ac_{2}\right]}_{100\%}}+\frac{0.50\times [CHXCarb]}{[Na_{2}]{\left[Carb\right]}_{100\%}}$$

### 3.7. Dissolution Profile Measurement

The dissolution rates for β-lactam-based chlorhexidine GUMBOS were measured using a method similar to Lengsfeld *et al.* (2002) [32]. Here, 20 mg GUMBOS were stirred 50 mL of deionized water. Over time, 1-mL aliquots were collected and filtered through a 0.1-µm syringe filter and analyzed spectrophotometrically at 260 nm in triplicate until the absorbance values approached a plateau.

### 3.8. In Vivo Prediction of Intestinal Permeability Coefficients and Absorption

Intestinal permeability was approximated using a protocol outlined in the BD Gentest Pre-coated PAMPA Plate System Assay (BD Biosciences, Bedford, MA, USA). Membrane permeability was calculated using formulas provided in the assay, and resultant predictive intestinal absorption values were determined using a log permeability coefficient ≤−6 threshold that estimates ≥75% intestinal absorption. Predictive intestinal absorption was analyzed by analysis of variance (ANOVA) with comparisons of means to check statistical significance using SAS 9.2 2 (SAS Institute Inc., Cary, NC, USA), *p* < 0.05.

### 3.9. Mammalian Cytotoxicity

*In* vitro experiments were performed using fibroblasts (NIH/3T3), endothelial (EOMA) and cervical (HeLa) cell lines (ATCC, Manassas, VA, USA) by Karen McDonough of the Veterinary Science Department at Louisiana State University using conventional cell viability methods. Cytotoxicities of chlorhexidine-based salts and their stoichiometric mixtures were determined using the MTT assay kit (Promega Corporation, Madison, WI, USA). Test compounds up to 500 µM were serially diluted directly into cell culture media and transferred to seeded cells (×10^9^). Each concentration was performed in triplicate. Cells were incubated for 24 h at 37 °C, in 5% CO_2_ atmosphere. At the end of the incubation period, cell viability was quantified at 570 nm in a microplate spectrophotometer (Benchmark plus Bio-Rad Laboratories, Hercules, CA, USA). Cell viability as a percentage was determined by computing the ratio between absorbance of the treated cells and the absorbance of untreated (control) cells taken as 100%. Reported values are the lethal concentrations able to kill 50% of the population of viable cells (LD_50_). Therapeutic indices were calculated by dividing the mean MIC value per group of microorganisms (*i.e*., drug susceptible Gram-positive bacteria, *MRSA*, drug susceptible GNB and multi-drug-resistant Gram-negative bacteria) into the LD_50_ for each cell type. Cytotoxicity results were analyzed by analysis of variance (ANOVA) with comparisons of means to check statistical significance using SAS 9.2 2 (SAS Institute Inc., Cary, NC, USA), *p* < 0.05.

## 4. Conclusions

The use of reacted ion pairs (GUMBOS) composed of previously approved drugs and outmoded antibiotics shows promise as an alternative combinatorial drug strategy for treating wound infections caused by drug-resistant bacteria. GUMBOS formed from β-lactam antibiotics and chlorhexidine diacetate were found to: (1) extend the spectra of antibacterial activity with profound antibacterial activity; and (2) lower the concentration required to inhibit the growth of multi-drug-resistant bacteria better than the unreacted, stoichiometric equivalent of precursor ions. Even more so, GUMBOS were less toxic to invasive cell types commonly found in superficial and chronic wounds. Overall, this approach may offer an alternative approach to contain ion-pairs and effectively execute the principles of combination drug therapy. Future studies include investigating mechanisms of action, identifying GUMBOS potential in mitigating other diseases for which combination drug therapy is commonly applied and evaluating drug pharmacokinetic behavior using *in vivo* animal models.
